# Predictors of Distribution and Diversity of Rare, Protected, and Endangered Freshwater Mollusks in Rivers With Various Land Use in the Context of Environmental Changes

**DOI:** 10.1002/ece3.71209

**Published:** 2025-04-15

**Authors:** Tomasz Krepski, Anna Cieplok, Aneta Spyra

**Affiliations:** ^1^ Department of Hydrobiology Institute of Biology, University of Szczecin Szczecin Poland; ^2^ Faculty of Natural Sciences Institute of Biology, Biotechnology, and Environmental Protection, University of Silesia Katowice Poland

**Keywords:** anthropopressure, catchments, diversity metrics, global changes, sustainability, water management

## Abstract

Rivers face multiple stressors that act on biodiversity. In view of global environmental changes, river modifications, deforestation, and pollution, their natural parts are of highest importance in maintaining the biodiversity of mollusks. The study addresses the predictors of rare mollusk species, as they serve as ecological indicators of ecosystem quality and drivers of ecosystem pollution in agricultural, urban, and forest catchments. Analyses of environmental predictors and mollusk distribution were provided, and the model of multivariate canonical correspondence analysis was constructed. The pH of the water was relatively high, indicating alkaline conditions, whereas conductivity was low, indicating no salty influences in river basins. The study revealed the occurrence of species globally threatened (EN), vulnerable in Europe (VU), least concern species (LC), or legally protected in Poland, for example, *Unio crassus, Anodonta cygnea*, and *Unio tumidus* . Mollusks showed a high positive association with 13 predictors that reflected the variety of substrates, river and water parameters, and debris. As shown by CCA results, they also avoided rivers in urban catchments. The results provide new data for managing areas, and the specifics are easy to extrapolate to other contexts. Mollusks occurrence in freshwaters has decreased dramatically, placing them among the most threatened animals on Earth. Overall, this study highlights the complex interactions between environmental conditions and mollusk diversity.

## Graphical abstract

Rivers face multiple stressors that act on biodiversity. In view of global environmental changes, river modifications, deforestation, and pollution, their natural parts are of the highest importance in maintaining the biodiversity of mollusks; therefore, the study addresses the predictors of rare species, as they serve as ecological indicators of ecosystem quality and drivers of ecosystem pollution in agricultural, urban, and forest catchments. Mollusks occurrence in freshwaters has decreased dramatically, placing them among the most threatened animals on Earth. Overall, this study highlights the complex interactions between environmental conditions and mollusk diversity.

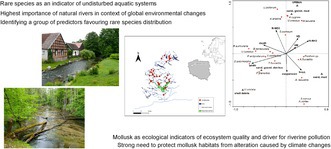

## 1 Introduction

The alterations in hydrological characteristics of rivers, including the magnitude and timing of high and low flows and thermal regimes, trigger ecological alterations in river ecosystem functioning. The study of river ecosystems highlights the ecological fragility and spatial heterogeneity of the risks that unmitigated climate change poses to river ecosystems (van Vliet et al. 2013; Thompson et al. 2021). Multiple stressors act simultaneously on the biodiversity of rivers (Reid et al. 2019), but following Feio et al. (2023), consequences are poorly quantified at the global scale. Various human pressures, including increased human populations and consumption (Reid et al. 2019), eutrophication due to excess nitrates and phosphates, and pollution discharges, alter river health and flow regimes (Belletti et al. 2020) and ecosystem functioning (Pereira and Ferreira 2021). River transformations include changes in morphology, hydrology, nutrients and toxic substances, ecosystem metabolism and the storage of carbon (C), loss of native species, expansion of invasive species, and disease emergence (Carpenter et al. 2011).

In view of river modifications, regulation of riverbeds, deforestation, and pollution in river catchments, natural parts of rivers are of special importance in maintaining biodiversity in aquatic invertebrate communities, especially mollusks. Morphological alterations of rivers affect hydrological conditions, including regulation of the riverbed (Bylak and Kukuła 2007; Pedersen et al. 2014), natural morphological transformations of the riverbeds (Garcia et al. 2012), the occurrence of natural (Arndt and Domdei 2011; Spyra et al. 2023) or artificial barriers (Martínez et al. 2013; Tiemann et al. 2004), or the transposition of rock or wood debris, which may lead to a depletion of the number of fauna taxa or, in some cases, conversely, to an increase in the number of taxa (Merz and Ochikubo Chan 2005; Braccia and Batzer 2008; Hrodey et al. 2008), significantly influencing the structure of benthic communities and also affecting the mollusk fauna.

Alterations of riverbed morphology caused by river damming have an impact on hydrological processes and biocenosis. (Growns et al. 2014; Ren et al. 2014; Van Looy et al. 2014; Kiraga and Remer 2023; Pařil 2023). Even a small dam formed on a stream causes a change in the nature of fauna communities from lotic to limnic above and below the barrier (Käiro et al. 2012). In various climatic conditions or different ecoregions, the impact of the same environmental factors on aquatic fauna, including mollusks, will be different. In rivers with riverbed transformation, decreasing species richness is observed due to the accumulation of organic matter above the dam and its transport below, which disturbs trophic structures, leading to the disappearance of sensitive taxa (Cortes et al. 1998; Wang et al. 2013). Hence, various changes at the ecological level have a completely different effect on benthic communities in upland streams than in lowland streams (Tszydel et al. 2009).

Freshwater snails and mussels are numerous groups of benthos, and they play a key role in the functioning of aquatic ecosystems (Garg et al. 2009). Being an important indicator of environmental changes, individual species respond directly or indirectly and sensitively to changes in chemical and physical parameters. Freshwater mollusks are used in biological monitoring studies to determine metal contamination and water quality as pollution indicators (Lee et al. 2002; Wadaan 2005; Fortunato 2015; Gümüş et al. 2022). Furthermore, their shells serve as shelters from predators and physical and physiological stress and form a substrate for periphytic organisms (Gutiérrez et al. 2003). Rivers serve as habitats for rare, endangered, and protected species of mollusks. However, their occurrence decreases due to the degradation of water systems, pollution, and river alteration (Zawal et al. 2016; Bănăduc et al. 2022). In light of ongoing climate change, such as climate warming, their populations are particularly threatened.

Studies conducted in river basins are principal in terms of their overall functioning, especially when studied in different catchments. The rivers studied can serve as a model structure to study predictors of the distribution and diversity of freshwater Mollusca in rivers with forest, urban, and agricultural catchment in this part of Europe in the context of global environmental changes. In this study, we used mollusks as an ecological indicator of the ecosystem quality and a driver for riverine ecosystem pollution. We followed by hypotheses that (1) the type of river catchment (agricultural, urban and forest) will have an impact on the structure of freshwater mollusk communities; (2) the anthropogenic impact (such as e.g., municipal and agricultural wastes discharges, shore littering, and recreational use) will differentiate the distribution of mollusks in the rivers located within different catchments; and (3) environmental factors will influence the occurrence of rare and protected species of mollusks as they serve as ecological indicators of ecosystem quality.

## 2 Methods

### 2.1 Study Area

**FIGURE 1:**
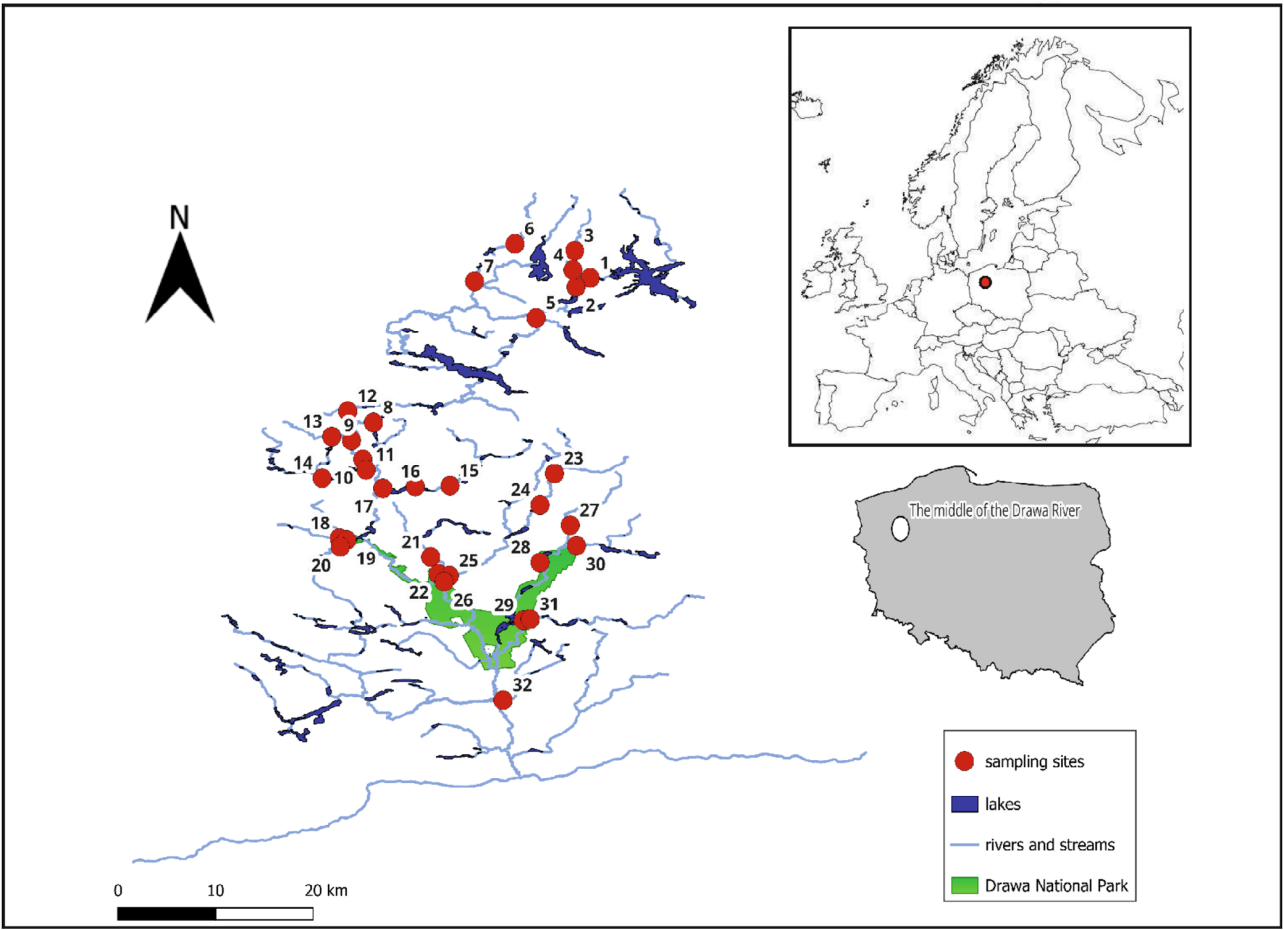
Location of the rivers studied in agricultural, urban, and forest catchments (Northern Poland).

The studied rivers are located in northwestern Poland (Figure 1) in the Drawa River Basin, whose sources are located at an altitude of 150 m above sea level near Połczyn Zdrój and the Valley of Five Lakes Nature Reserve. The studied rivers and their tributaries serve as a unique natural area; therefore, to preserve its natural environmental values, two protected areas have been established—the Drawski Landscape Park, which protects part of the upper catchment area, and the Drawa National Park, encompassing the middle Drawa and the middle and lower parts of the Płociczna River (Przybylski et al. 2020). This territory is extensively forested. Forests cover 949.2 km^2^ of area, corresponding to 29.7% of the basin's total area. Rivers are located partly in the Drawa National Park (DNP) territory in an area of 11,019 ha. The watercourses located in different catchments (Table S1) are characterized by different anthropopressure intensities (Domagała et al. 2010), various hydrological conditions, and different catchment management. Agricultural areas characterize the northern part of this area, and a relatively sizable anthropogenic impact is observed. The central part of the studied area is located partly within the boundaries of the DNP, is forested, and, taking into account the presence of the National Park and a military traverse, has a smaller anthropogenic impact than other sections. In the DNP area, a high tourist pressure on rivers is observed, for example, in the Korytnica and Drawa rivers (Domagała et al. 2010). The southern part of the studied region is located through agricultural wastelands and forests. In some of the rivers studied, numerous artificial dams, bed regulations or reinforcements, and beaver dams (Table S1) are present. In this study, rivers with natural river beds and no anthropogenic transformation and pressure were also selected as well as the rivers with minimally modified sections.

### 2.2 Sampling and Laboratory Analyses

The study was conducted across 32 sections of rivers with varying catchments, anthropogenic transformations, or impacts during two sampling seasons (Spring and Autumn). Sampling involved using a modified “Kajak” tubular sampler (Blomqvist and Abrahamsson 1985), with an 8 cm diameter (to 25 cm deep), which was equipped with serrations on the lower rim to facilitate the collection of sediment fragments from sand and gravel bottoms. At each site, a 100 m transect was established, from which three samples were taken, each consisting of 6 cores with a volume of 1256 cm^3^ each, covering a total area of 301.44 cm^2^. Sample locations were chosen based on common microhabitats, with each sample serving as a repetition. After collection, samples were strained through a 0.25 mm mesh, decanted, and preserved in a 95% ethyl alcohol solution before transportation to the laboratory. Mollusks were then selected and identified using a Nikon SMZ 745 T stereoscopic microscope, followed by fixation in a 70% ethyl alcohol solution.

River width and depth were measured at each site at three points, and water velocity was measured at three levels: at the bottom, halfway between the bottom and the surface, and near the surface to obtain an average water velocity. Velocity measurements were taken using a SENSA Z300 electromagnetic water velocity sensor with velocity calculated based on results from measurements such as the width and the depth of the stream in the hydrometric verticals. Water velocity was measured in the bottom area (VD) and at a depth of 60% (VS). Physicochemical water parameters, including temperature, conductivity, pH, and dissolved oxygen concentration, were measured using a HydroLAB DS probe. Water samples were also collected for laboratory analysis of nitrite nitrogen (N–NO_2_), nitrate nitrogen (N–NO_3_), ammonium nitrogen (N–NH_4_), total nitrogen (NTOT), phosphates (P–PO_4_), total phosphorus (PTOT), and water suspension, using a HACH LANGE DR850 colorimeter.

Additionally, each site was detailed regarding anthropogenic threats (such as, municipal, industrial, and agricultural wastes, shore littering, and recreational use) shoreline type (natural untransformed riverbed, riverbed damming and regulation shore reinforcements, presence of fascines and water threshold), and surrounding stream development. The bottom grain size was examined by randomly collecting three 1256 cm^3^ cores along a 100 m transect at each river section, using the same sampler as for mollusk sampling. Samples were placed in string bags, transported to the laboratory, and processed by decanting organic matter and drying the remaining material. The dried samples were then sieved through meshes of various sizes (63, 16, 2, 0.5, 0.25, and 0.063 mm) for analysis.

### 2.3 Zoocenological and Statistical Analyses

The composition of the mollusk community was evaluated using various methods, including the dominance index (D) [%] (Biesiadka and Kowalik 1980), which categorizes species as eudominants (D > 10%), dominants (D = 5.1%–10%), subdominants (D = 2.0%–5.0%), and recedents (D < 2.0%). The relative abundance of species [%] was expressed as the percentage composition of specific benthic taxa relative to the total number of collected specimens. The Shannon–Wiener diversity index (H′) and Simpson diversity index were also calculated. These diversity indices were computed using the MVSP program version 3.13p (Kovach Computing Services). To investigate faunistic similarities in mollusk communities in rivers with different catchment types, an HCA (Hierarchical Cluster Analysis) was conducted. In the HCA approach, the distance between clusters was measured using the Euclidean distance metric according to Ward's method. This analysis was performed using the MVSP 3.13.p Software from Kovach Computing Services. The population trend was defined according to The IUCN Red List of Threatened Species (IUCN 2024) and Cuttelod et al. (2011). Differences in physical and chemical water parameters, velocity, depth, and width of the rivers, as well as the density between the two sampling seasons, were tested using Mann–Whitney *U*‐tests. This statistical analysis was conducted using STATISTICA version 13.1 from Dell.

Thirty two Sampling sites were grouped into the different disturbance categories (slight, moderate, and severe) or land use groups (agricultural, urban, and forest). The significance of the differences in the land use, type of disturbances, and the density of mollusks between the river sections was evaluated using the ANOVA Kruskal‐Wallis and multiple comparisons post hoc test. The data was found to be of a non‐normal distribution (Kolmogorov–Smirnov test for normality) (STATISTICA version 13.1; Dell version).

The influence of environmental variables, such as physicochemical parameters of water, velocity, sediment composition, catchment types, anthropogenic influence, and riverbed transformations on the structure of mollusk communities was assessed using canonical correspondence analysis (CCA) (Ter Braak 1986) with CANOCO software version 4.5. The data sets with absolute values of community components were first subjected to detrended correspondence analysis (DCA) to reveal prevailing patterns of response curves in relation to environmental gradients (the length of the gradient was 3.354). The significance of the axes generated in the analysis was validated through the Monte Carlo test (Ter Braak and Smilauer 1998). Gradient analyses were initially performed with all environmental variables, followed by forward selection to extract the variables that significantly contributed to the ordination. Considering the response and explanatory variables, the ordination diagram was created using CanoDraw (CANOCO software version 4.5) based on statistically significant variables. A *p*‐value of < 0.05 was considered to be significant.

## 3 Results

The analysis of physical and chemical water parameters revealed that the pH of rivers was high, indicating alkaline waters, with a range of 7.58–9.80 (Table S2). Conductivity was relatively low, ranging from 172 to 464 μS/cm, indicating the absence of saline influences in the river basin. Despite agricultural catchments in some rivers, the total nitrogen values were relatively low, as were N‐NO_3_ (0–3.9 mg/L) and N‐NO_2_ (0–0.078 mg/L) concentrations (Table S2). Phosphate values ranged from 0.14 to 4.49 in the studied rivers. Statistical analyses showed significant differences in dissolved oxygen, pH, conductivity, N–NO3, N–NO2, N–NH3, Ptot, and suspension values between the sampling seasons (U Mann–Whitney test; O_2_Z = 4.578, *p* = 0.000005; pHZ = 6.854, *p* = 0.0000; CondZ = −2.175, *p* = 0.0296; N–NO_3_Z = 2.561, *p* = 0.0080; N–NO_2_Z = −2.363, *p* = 0.0181, N–NH_3_Z = −5.525 *p* = 0.0000, PTOTZ = −2.155, *p* = 0.0311; Suspension *Z* = 5.397, *p* = 0.0000).

In the studied rivers, 20 species of mollusks were found. Among the 11 species of snails, four species were classified as eudominants in some rivers: *Potamopyrgus antipodarum* , *Acroloxus lacustris, Ancylus fluviatilis*, *Bithynia tentaculata* , and *Theodoxus fluviatilis* . In the snail communities, all species are categorized as LC on a global scale. No species protected by law or rare species were detected. Population trends vary, with some species decreasing (e.g., *Viviparus contectus*) and stable (e.g., *Theodoxus fluviatilis* ) or increasing in Europe (e.g., *Potamopyrgus antipodarum* ) (Table S3). In mollusk communities, two alien and invasive species were found: *D. polymorpha* and *P. antipodarum* .

*Pisidium* sp. was the most numerous in the bivalve fauna. The eudominant group included *Sphaerium corneum* and *Dreissena polymorpha* , while the dominant group included *Unio tumidus* and *Anodonta anatina* . Seven species of bivalves in this study are classified as least concern (LC) on a global scale, and one species *Unio crassus* is classified as endangered (EN). *Unio crassus* is also listed in the Polish Red List of Species as EN (Table S3). Population trends (decreasing, increasing, unknown, stable) for all bivalve species vary but are the same on a global and European range. Three species of bivalves have decreasing population trends: *U. crassus* , *A. anatina* , and *Anodonta cygnea* . *Dreissena polymorpha* has an increasing population trend, while the population trends of the other species remain unknown (Table S3). We found the occurrence of one species protected by Polish Law ( *Unio crassus* ) and one species partially protected ( *Anodonta cygnea* ).

**FIGURE 2:**
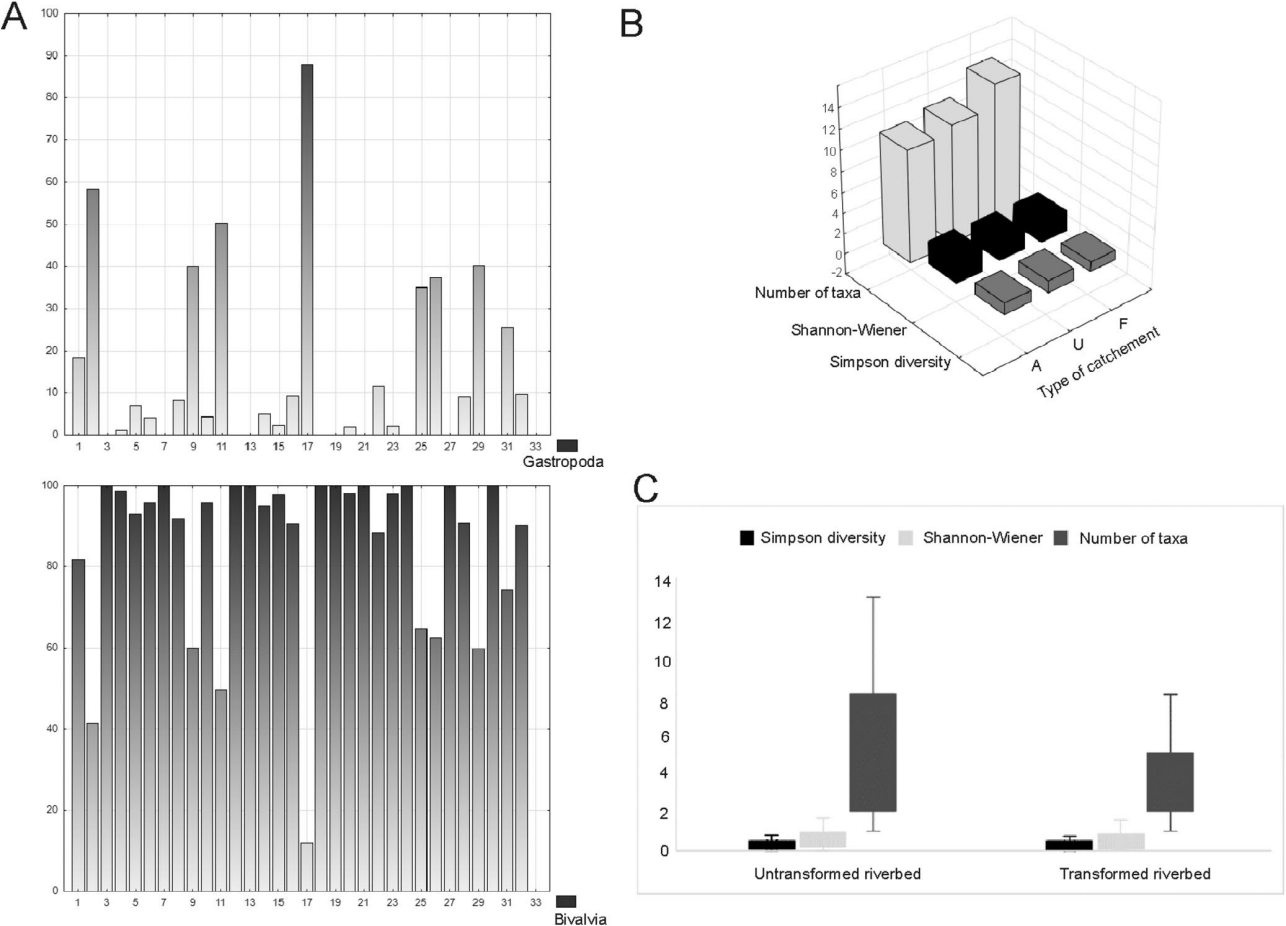
(A) The relative abundance of Gastropoda and Bivalvia in the rivers. 1–33: Rivers studied, numbering as in Online Resources S1. (B) The diversity indices and number of taxa in rivers of agricultural (A), urban (U), and forest (F) catchments. (C) The diversity of Mollusk communities in rivers with natural, untransformed riverbed and riverbed regulation, reinforcements, and damming.

**FIGURE 3:**
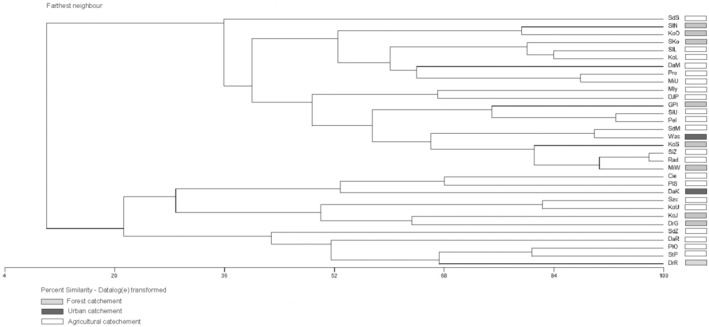
The results of hierarchical cluster analysis of faunistic similarities in the rivers studied: Cie, Cieszynka; DaK, Drawica Kalisz; DaM, Drawica Mąkowary; DaR, Drawica Rościn; DJP, Dopływ J. Pańskie; DrG, Drawa Głęboczek; DrR, Drawa Rzepowo; GPl, Górna Płociczna; KoJ, Korytnica Jaźwiny; KoL, Kokna Las; KoO, Kokna Ostrowice; KoS, Korytnica Studnica; KoU, Korytnica Ujście; MiU, Miedźnik Ujście; MiW, Miedźnik Nowe Worowo; Mly, Młynówka; Pel, Pełknica; PlO, Płociczna Ostrowieckie; PlS, Płociczna Sitno; Pro, Prosta; SdM, Stara Drawa Mostek; SdS, Stara Drawa Środek; SdZ, Stara Drawa Zapora; SiU, Sitna Ujście; SiZ, Sitna Zapora; SKo, Stara Korytnica; SlL, Słopica Leśniczówka; SlN, Słopica Niemieńsko; StP, Stary Potok Rad Radówka; Szc, Szczuczna; Was, Wąsówka.

Hierarchical cluster analysis (HCA) of faunistic similarities confirmed differences in mollusk communities between rivers in the Drawa River Basin. In the upper part of the diagram, rivers where mussels constituted 95%–100% of the collection (15 out of 20 rivers) were grouped, with a small percentage of freshwater snails found in their fauna (Figure 2A, Figure 3). In the lower part of the diagram, 12 rivers were grouped into one agglomerate, with numerous occurrences of mussels and a relatively high percentage of snails ranging from 8.22% to 88.04% (Figure 2A, Figure 3).

The values of diversity indices, both Simpson and Shannon–Wiener, were similar regardless of river catchment type, but the number of mollusk species was highest in rivers located within forest catchments. Analysis of river transformation and diversity indices showed that values in anthropogenically modified and natural river sections are similar, indicating minor changes in the riverbed, but the average number of taxa was higher in natural and untransformed river sections (Figure 2B, Figure 2C). Results of ANOVA showed differences in the occurrence of mollusks in the river section with different disturbances (slight, moderate, and severe) as well as the different land use (agricultural, urban, and forest) were statistically insignificant (ANOVA KW: H (2, *N* = 32) = 0.2438672 *p* = 0.8852 and H (2, *N* = 32) = 0.2454545 *p* = 0.8845, respectively). Statistical analyses showed insignificant differences in the density of mollusks in the studied seasons (*Z* = −148,702, *p* = 0,1370).

**FIGURE 4:**
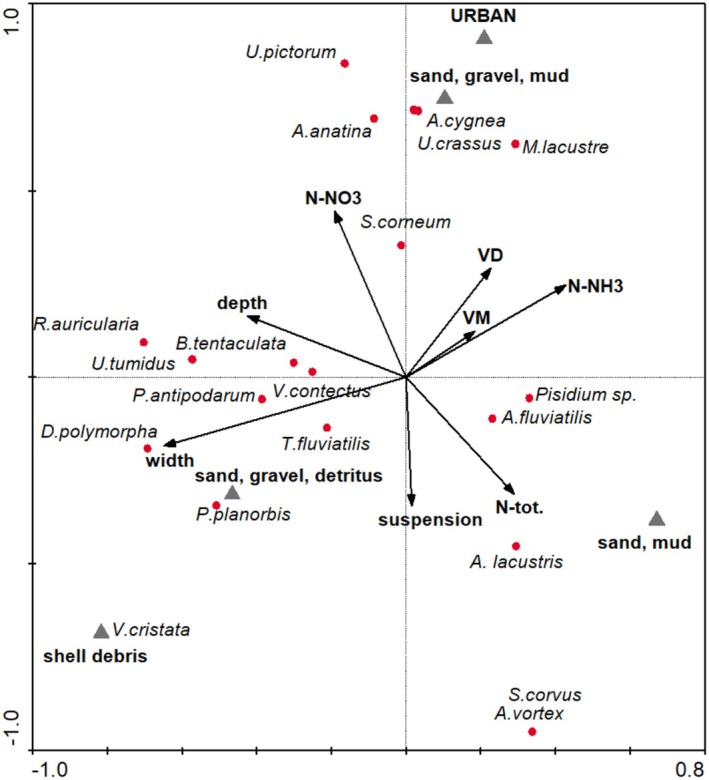
Canonical correspondence analysis diagram showing the gastropod and bivalve communities in relation to environmental parameters in rivers.

In the CCA analysis, mollusk communities showed a high positive association with thirteen variables reflecting substrate variety, river width and depth, velocity, urban river catchment, and different water parameters (*p* < 0.05) (Table 1, Figure 4). The contribution of these variables to species variability was found to have a statistically significant effect (Table 1). CCA (on species and environmental data) showed that the first and second axes explained 28.5% of the total variation in the species data and 42.7% of the variance in the species and environment relationship (Table S4). Mollusk species located on the right side of the ordination diagram were mostly associated with rivers with higher values of N–NH_3_ (0–1.40 mg/L), NTOT (0.2–10.7 mg/L), suspension in the water, and velocity. *Unio crassus* and *A. cygnea* were mainly associated with sand and mud with gravel sediments. Species located on the left side of the ordination space were associated with river depth and width, N–NO_3_ concentration in the water, and bottom sediments covered with debris, while they tended to avoid rivers with urban catchments. The occurrence of *T. fluviatilis* , *P. planorbis*, *Viviparus contectus*, and *D. polymorpha* was related to bottom sediments covered with detritus. One species, *Valvata cristata*, occurred in rivers with shell debris covering the bottom sediments. The Monte Carlo test confirmed the statistical significance of these results (*p* = 0.0020) (Table S4).

**TABLE 1:** Results of forward selection and Monte Carlo permutation tests from canonical correspondence analysis (CCA).

Parameter	λ‐1	λ‐A	*p*
Width	0.18	0.18	0.006
Sand, gravel, mud	0.15	0.15	0.002
VM	0.10	0.12	0.004
VD	0.10	0.10	0.010
Depth	0.14	0.11	0.004
Sand, gravel, detritus	0.12	0.09	0.034
N‐NO3	0.08	0.09	0.010
N‐tot.	0.12	0.07	0.010
Shell debris	0.12	0.07	0.042
N‐NH3	0.15	0.06	0.040
Suspension	0.02	0.06	0.042
Urban	0.07	0.06	0.044
Sand. mud	0.04	0.05	0.020

## 4 Discussion

The progressive anthropogenic pressure caused by protracted agricultural and urban activity has a negative effect on river systems worldwide. Brauns et al. (2022) revealed the risk of losing the functional backbone of rivers and the critical ecosystem services they provide if human stressors persist at current intensities. In light of that, studies covering entire river basins, such as this study, are crucial. They reveal the occurrence of mollusk communities with rare, protected, and endangered species and help identify predictors of their distribution and diversity. Given global environmental changes, the occurrence of such species is particularly at risk. Climate change scenarios predict changes in runoff over the next century if global warming of 2°C–4°C occurs (Carpenter et al. Please provide the sentences you would like me to rewrite according to the specified guidelines. 1992; Donnelly et al. 2017). Flaschka et al. (1987) found that in arid regions, drier conditions will decrease water supply rates below current human demand. Water systems, along with land transformations for agricultural use, are among the most extensively and rapidly altered ecosystems on Earth (Carpenter et al. 2011). Climate change is also projected to increase the frequency and magnitude of extreme events such as floods and droughts (IPCC 2013), which will further impact aquatic mollusk fauna.

Anthropogenic damming of rivers leads to radical changes in their hydrology, especially water velocity and flow. The relationship between water velocity and flow and species composition in aquatic fauna communities is a frequently discussed issue in hydrological impact studies (Rolls et al. 2012; Korte 2010; Campbell et al. 2015; Brysiewicz et al. 2022). Increased water flow tends to increase the number of rheophilic taxa, such as a representative of the Neritidae family— *T. fluviatilis* (Gregs et al. 2014). However, with the decrease in water velocity, an increase in the number of limnic taxa is observed, such as freshwater snails, for example, *Stagnicola palustris* (Strzelec and Królczyk 2004). This study confirms these findings, as velocity significantly influenced mollusk communities in the studied rivers. Parameters such as water chemistry and river transformations also have a significant impact on aquatic communities, as evidenced by the association of mollusk communities with 13 variables that reflected the variety of substrates, the river width and depth, urban river catchments, and water parameters such as N–NH_3_, NTOT, suspended solids, and velocity. The type of bottom sediments has an impact on the occurrence of *T. fluviatilis* , *P. planorbis*, *V. contectus*, and *D. polymorpha* , which were related to bottom sediments covered with detritus, while *V. cristata* was associated with shell debris covering the bottom sediments. It proves that predictors like water chemistry and river transformations have an equally significant impact on aquatic communities in rivers. Hydrological changes in rivers can lead to changes in physical and chemical parameters, which in turn influence the trophic status of the aquatic environment. The concentration of biogenic elements, particularly phosphorus and nitrogen, plays a crucial role in determining the trophic conditions of the environment for aquatic organisms. Some studies suggest that relatively high concentrations of phosphorus in water negatively affect the biodiversity of aquatic fauna, similar to nitrogen compounds (Ashton et al. 2014; Santoro et al. 2014; Thanigaivel et al. 2023). Despite agricultural catchments in some rivers, our study found relatively low values of total nitrogen, N–NO_3_, and N–NO_2_. Phosphate values ranged from 0.14 to 4.49 in the studied rivers. The pH of rivers was high, indicating alkaline waters, ranging from 7.58 to 9.80, while conductivity was relatively low, ranging from 172 to 464 μS/cm, indicating the absence of saline influences in the river basin. All these parameters showed significant differences between the sampling seasons, similarly to our previous study (Cieplok and Strzelec 2018; Krodkiewska et al. 2022).

Considering the impact of various environmental factors on the dynamics and functioning of the freshwater mollusk fauna, it is evident that significant progress has been made in understanding these relationships. However, comprehensive research on river ecosystems remains a challenge for modern ecohydrologists seeking to understand the relationships between environmental conditions and mollusk communities. Many of them claim that the greatest influence on the structure of aquatic macroinvertebrates is the nature of the immediate environment of the river in which they occur (Collier and Clements 2011; Guellaf et al. 2023); others highlight the conditions of the catchment area or even climatic factors on assemblage structures (Bis et al. 2000). The nature of the catchment area, particularly its use, is a crucial aspect influencing mollusk fauna. Rivers running through forests or agricultural wasteland are often characterized by higher biodiversity or taxonomic richness of invertebrates compared to those in urbanized or agricultural areas (Genito et al. 2002; Couceiro et al. 2007; Muehlbauer and Doyle 2012). Therefore, it was important to closely examine these anthropogenic factors, such as transformations in the stream environment, which directly affect water quality and the qualitative and quantitative structures of mollusk communities in studied rivers.

Mollusks are the most diverse and widespread aquatic animals in freshwater systems (Böhm et al. 2021). However, their occurrence has decreased dramatically, leading to their classification as some of the most threatened animals on Earth (Lopes‐Lima et al. 2021). Many species are already extinct or on the brink of extinction. This study revealed the occurrence of rare and valuable mollusk species in river ecosystems, including *U. crassus* , which is globally endangered (VU in Europe) (IUCN 2024). The population of *U. crassus* is decreasing due to numerous threats, including agriculture and aquaculture, such as annual and perennial non‐timber crops and wood and pulp plantations. This species is endangered due to natural system modifications, dams and water management, invasive species, genes, and diseases (Tomović et al. 2023). Domestic and urban wastewater, agricultural and forestry effluents, nutrient loads, soil erosion, and sedimentation are crucial in the decreasing occurrence of *U. crassus* (Patzner and Müller 2001). Conservation actions such as protecting the sites of its occurrence and monitoring population and habitat trends are necessary due to global climate changes and habitat loss (Lopes‐Lima et al. 2014; Beran 2019; Soroka et al. 2021). The density of *U. crassus* populations has declined dramatically on the territory of Europe during the second half of the 20th century (Denic et al. 2013), which led to the annexing of this species in the II and IV Council Directive 92/43/EEC (1992). The multivariate analysis conducted in the studied rivers revealed that *U. crassus* and *A. cygnea* were mainly associated with sand and mud with gravel sediments. Other mollusk species were primarily associated with river depth and width, N–NO_3_ concentration in the water, and bottom sediments covered with debris, while they tended to avoid rivers with urban catchments.

Following the IUCN Red List category, *U. tumidus* is assigned to LC. However, specific threats such as residential and commercial development, natural system modifications, invasive and other problematic species, genes and diseases, and pollution from domestic and urban wastewater, industrial and military effluents, and agricultural and forestry effluents (IUCN 2024). The species population trend remains unknown, but considering the decline in European bivalve communities (Bogan 2008; Lopes‐Lima et al. 2017), it should be stated that the trend may not be increasing. Therefore, there is a strong need for research on population size and distribution to assess its global trend.

*Anodonta cygnea* , globally categorized as LC in the IUCN Red List, is partially protected by Polish Law. However, its population trend is decreasing due to threats such as residential and commercial development, including housing urban areas, commercial and industrial areas, tourism and recreation areas, and agriculture and aquaculture; natural system modification, such as annual and perennial non‐timber crops (IUCN 2024). Like other species, *A. cygnea* habitats are also vulnerable to natural system modifications in the form of dams and water management as well as other ecosystem modifications (Piechocki and Dyduch‐Falniowska 1993; Byrne et al. 2009), pollution of river ecosystems, such as those from domestic and urban wastewater, as well as climate change and severe weather, for example droughts (Lopes‐Lima et al. 2021). Due to the decline of mollusk species resulting from these environmental risks, monitoring studies of their distribution, population size, and trends are strongly recommended, especially for globally threatened and data‐deficient species. Research indicates that mollusks exhibit similar chemical sensitivities in aquatic assemblages with similar taxonomic compositions, but those with different compositions could vary slightly or greatly (Liang et al. 2024). Therefore, efforts to protect river catchments, preserve and restore forested catchments, treat wastewater discharges, and improve river connectivity are crucial for the conservation of mollusk species (Feio et al. 2023).

The multifaceted types of global human impacts have led to the emergence of “The Anthropocene,” an era characterized by unprecedented pressure on Earth's natural ecosystems, where human impact dominates fundamental processes (Malhi 2017; Arthington et al. 2023). The need for action to protect the integrity and biodiversity of river systems has become increasingly urgent, prompting significant conservation and restoration efforts (Lynch et al. 2023). Global changes can impact ecological communities differently across gradients as they are exposed to various environmental stressors, leading to warmer and more anthropogenically modified habitats (Trew and Maclean 2021; Woods et al. 2023). This is especially important because many regions are home to rare, vulnerable, and functionally unique taxa that may be disproportionately sensitive to global change (Myers et al. 2000). The results of this study have implications for environmental protection strategies and improving the sustainability of the ecosystem. The novelty of our study is to provide baseline and ecological predictors in river basins in water resource planning and management. Our findings bring novelty to the study of mollusk occurrence and dynamics by evidencing the patterns of occurrence of environmental predictors found in this study.

## 5 Conclusions and Protection Priority

The type of river catchment partially impacted the structure of freshwater mollusk communities. The number of mollusk species was the highest in rivers located in forest catchments; mollusk populations do not colonize rivers in urban catchments. Analysis of the transformation of the river and the diversity indices showed that their values in anthropogenically modified and natural river sections are similar, which indicates a minor anthropogenic impact in the riverbed on the distribution of mollusk fauna. However, the average number of taxa was higher in the natural and untransformed river sections.

Mollusks showed a high positive association with thirteen predictors that reflected the variety of substrates, the river width and depth, velocity, urban river catchment, and different water parameters such as N‐NH_3_, NTOT, suspension, and velocity. *Unio crassus* and *A. cygnea* were mainly associated with sand and mud with gravel sediments. The occurrence of the largest number of species was associated with the depth and width of the river, N‐NO_3_ concentration in the water, and bottom sediments covered with debris. The results provide important new information for understanding and managing areas, and the specifics are easy to extrapolate to other contexts. Human activities like water pollution, recreation, or riverbed transformation have an impact on the river ecosystems, posing a risk to the delicate ecological balance. Therefore, all river catchments should be under specific management or protection, especially focused on the protection of habitats of rare species.

## Author Contributions

**Tomasz Krepski:** conceptualization (equal), data curation (equal), investigation (equal), methodology (equal), project administration (equal), writing – original draft (equal). **Anna Cieplok:** formal analysis (equal), writing – original draft (equal), writing – original draft (equal), writing – review and editing (equal), writing – review and editing (equal). **Aneta Spyra:** formal analysis (equal), writing – original draft (equal), writing – review and editing (equal).

## Conflicts of Interest

The authors declare no conflicts of interest.

## Supporting information


Appendix S1.

## Data Availability

The data generated during this study are available as a Appendix S1.
