# Supplementary material for: Predictors of Distribution and Diversity of Rare, Protected, and Endangered Freshwater Mollusks in Rivers With Various Land Use in the Context of Environmental Changes
Source: Ecol Evol. 2025 Apr 15;15(4):e71209. doi: 10.1002/ece3.71209 (PMC11997464; doi:10.1002/ece3.71209)
Supplement: Supplementary file 1 — Appendix S1. [file ECE3-15-e71209-s001.docx]

**ONLINE RESOURCES**

**SUPPLEMENTARY MATERIAL**

**Predictors of distribution and diversity of rare, protected, and endangered freshwater molluscs in rivers with various land use in the context of environmental changes**

Krepski T.^1^, Cieplok A.^2^, Spyra A.^2^*

^1^Department of Hydrobiology, Institute of Biology, University of Szczecin, Wąska 13, 71-712 Szczecin, Poland.

^2^Faculty of Natural Sciences, Institute of Biology, Biotechnology and Environmental Protection, University of Silesia, 9 Bankowa Str, 40-007 Katowice, Poland; *Corresponding author

E-mail address of the corresponding author: [aneta.spyra@us.edu.pl](mailto:aneta.spyra@us.edu.pl)

*corresponding author

e-mail address: [aneta.spyra@us.edu.pl](mailto:aneta.spyra@us.edu.pl)

Faculty of Natural Sciences,

Institute of Biology, Biotechnology and Environmental Protection,

University of Silesia,

Bankowa 9, 40-007 Katowice

Poland

**Table S1**

Characteristics of the rivers with various catchments. *- locality below damming, **locality in Drawieński Traverse, ***- locality in the protected area of Drawieński National Park.

| **River** | **Site** | **Locality in Drawa basin** | **Bottom sediments** | **Anthropogenic impact, pollution discharges** | **River management** | **Catchement type** |
| --- | --- | --- | --- | --- | --- | --- |
| Drawa | 1 | Upper course * | Shell debris | No anthropogenic impact | Natural untransformed riverbed | Agricultural |
|  | 2 | Upper course | Sand, gravel | - | Riverbed damming | Agricultural |
| Miedźnik | 3 | Upper course | Sand | - | Riverbed regulation | Agricultural |
|  | 4 | Upper course | Sand, mud | Municipal wastes discharges, agricultural wastes | Riverbed regulation | Forest |
| Wąsówka | 5 | Upper course | Mud, sand | Municipal, industrial wastes, rainwater from  city, shore littering | Riverbed regulation, shore reinforcements | Urban |
| Kokna | 6 | Upper course | Sand, mud | - | Riverbed regulation, fascines | Agricultural |
|  | 7 | Upper course | Sand, gravel, mud | - | Riverbed regulation | Forest |
| Stara Drawa | 8 | Middle course* | Mud | No anthropogenic impact | Natural untransformed riverbed | Forest |
|  | 9 | Middle course** | Mud, sand, gravel, wood debris | No anthropogenic impact | Natural untransformed riverbed | Forest |
|  | 10 | Middle course** | Sand, gravel, detritus | No anthropogenic impact | Natural untransformed riverbed | Forest |
| Stary Potok | 11 | Middle course | Sand, gravel, detritus | No anthropogenic impact | Natural untransformed riverbed | Forest |
| Radówka | 12 | Middle course** | Sand, gravel | No anthropogenic impact | Natural untransformed riverbed | Forest |
| Pełknica | 13 | Middle course** | Sand, gravel | No anthropogenic impact | Natural untransformed riverbed | Forest |
| Tributary L. Pańskie | 14 | Middle course | Sand, mud, detritus | No anthropogenic impact | Natural untransformed riverbed | Forest |
| Drawica | 15 | Middle course | Sand, gravel, mud | - | Riverbed regulation, fascines | Urban |
|  | 16 | Middle course* | Sand, gravel | - | Small dam, remnant of the mill | Forest |
|  | 17 | Middle course | Sand, gravel | No anthropogenic impact | Natural untransformed riverbed | Forest |
| Sitna | 18 | Middle course* | Sand, gravel | - | Water threshold before the mouth of stream | Forest |
|  | 19 | Middle course* | Sand, gravel | No anthropogenic impact | Natural untransformed riverbed | Forest |
| Prosta | 20 | Middle course | Sand, gravel | - | Riverbed regulation, concrete reinforcements | Forest |
| Słopica | 21 | Middle course* | Sand, gravel, mud | Municipal pollution, riverbed littering | - | Agricultural |
|  | 22 | Middle course | Gravel, sand | No anthropogenic impact | Natural untransformed riverbed | Forest |
| Korytnica | 23 | Upper course | Sand, gravel | Negligible impact of anthropopressure | Natural untransformed riverbed | Agricultural |
|  | 24 | Upper course | Sand, gravel | No anthropogenic impact | Natural untransformed riverbed | Agricultural |
|  | 25 | Upper course | Sand, gravel | - | Riverbed damming | Agricultural |
|  | 26 | Upper course | Gravel, sand | Tourism, canoeing | Natural untransformed riverbed | Forest |
| Płociczna | 27 | Lower course | Gravel, sand | No anthropogenic impact | Natural untransformed riverbed | Agricultural |
|  | 28 | Lower course*** | Sand, gravel, mud | No anthropogenic impact | Natural untransformed riverbed | Forest |
|  | 29 | Lower course*** | Sand, gravel shell debris | No anthropogenic impact | Natural untransformed riverbed | Forest |
| Młynówka | 30 | Lower course*** | Sand, gravel, mud | - | Small dam, remnant of the mill | Forest |
| Cieszynka | 31 | Lower course*** | Sand, gravel, mud, wood debris | No anthropogenic impact | Riverbed transform by beaver’s activity | Forest |
| Szczuczna | 32 | Lower course* | Sand, gravel | No anthropogenic impact | Natural untransformed riverbed | Forest |

**Table S2**

The results of the physicochemical analysis of the water in the rivers with various catchments (min-max), the river's characteristics, and velocity. VD - velocity measured in the bottom area; VS - Velocity measured at a depth of 60%; Conduct.- conductivity; Sus- suspension

| River | Site | O2  mg/L | pH | Conduct.  µS/cm | N-NO3  mg/L | N-NO2  mg/L | N-NH3  mg/L | N_TOT_  mg/L | PO_4_  mg/L | P_TOT_  mg/L | Sus | V_D_ | V_S_ | River width | River depth |
| --- | --- | --- | --- | --- | --- | --- | --- | --- | --- | --- | --- | --- | --- | --- | --- |
| Drawa | 1 | 7.52-11.01 | 8.42-9.80 | 209.1-212.4 | 0.1-0.2 | 0.005-0.003 | 0.03-0.36 | 2.4-10.7 | 0.37-0.66 | 0.09-0.34 | 4-14 | 0.08-0.10 | 0.21-0.37 | 7.4-9.4 | 0.7-0.9 |
|  | 2 | 8.66-10.16 | 8.47-9.29 | 216.4-238.7 | 0.2-0.2 | 0-0.012 | 0.01-0.31 | 1.9-3.0 | 0.39-0.40 | 0.12-0.30 | 4-35 | 0.06-0.23 | 0.37-0.57 | 8.1-10.8 | 0.4-0.7 |
| Miedźnik | 3 | 9.01-11.35 | 7.58-9.11 | 172.5-464.0 | 0.6-1.0 | 0 -0.018 | 0.33-0.47 | 5.1-6.0 | 0.37-0.60 | 0.16-0.22 | 2-18 | 0.18-0.49 | 0.29-0.61 | 1.9-2.4 | 0.1-0.3 |
|  | 4 | 9.00-9.30 | 7.61-9.14 | 205.7-704.0 | 0.5-0.5 | 0.003-0.031 | 0.39-0.46 | 6.0-6.9 | 0.31-0.70 | 0.19-0.24 | 5-15 | 0.03-0.14 | 0.23-0.36 | 3.2-4.1 | 0.1-0.3 |
| Wąsówka | 5 | 6.07-7.00 | 8.29-9.51 | 239.3-247.7 | 0.4-0.4 | 0.007-0.021 | 0.06-0.57 | 2.3-4.7 | 0.21-0.56 | 0.20-0.43 | 26-26 | 0.01-0.10 | 0.19-0.30 | 3.3-4.2 | 0.5-0.6 |
| Kokna | 6 | 5.48-8.05 | 8.83-8.86 | 201.3-255.2 | 0.3-0.3 | 0.010-0.011 | 0-0.34 | 2.3-4.2 | 0.30-0.68 | 0.18-0.42 | 4-21 | 0.04-0.27 | 0.37-0.52 | 6.1-8.0 | 0.4-0.6 |
|  | 7 | 9.30-11.42 | 7.45-9.15 | 210.5-313.0 | 0.1-0.6 | 0.003-0.006 | 0.35-0.47 | 3.5-3.8 | 0.44-0.74 | 0.20-0.24 | 3-9 | 0.02-0.14 | 0.08-0.32 | 3.1-3.8 | 0.2-0.6 |
| Stara Drawa | 8 | 6.66-8.92 | 7.86-9.40 | 297.0-238.4 | 0.3-1.6 | 0.006-0.009 | 0.03-0.42 | 1.4-3.8 | 0.14-0.22 | 0.12-0.25 | 3-14 | 0.01-0.02 | 0.00-0.06 | 6.9-10.0 | 0.3-0.4 |
|  | 9 | 6.27-8.87 | 7.84-9.13 | 269.0-270.0 | 0.1-0.4 | 0.003-0.007 | 0.35-0.45 | 5.1-9.9 | 0.39-0.75 | 0.19-0.30 | 5-6 | 0.02-0.05 | 0.08-0.15 | 7.0-10.9 | 0.4-0.4 |
|  | 10 | 8.06-9.58 | 8.20-8.96 | 188.1-216.0 | 0.1-0.6 | 0.002-0.004 | 0.04-0.31 | 1.4-2.2 | 0.71-1.02 | 0.10-0.41 | 4-22 | 0.15-0.32 | 0.49-0.54 | 9.3-13.6 | 0.3-0.4 |
| Stary Potok | 11 | 7.22-9.48 | 8.55-9.30 | 241-253.1 | 0.1-1.3 | 0.009-0.017 | 0.02-0.44 | 2.9-3.1 | 0.22-0.66 | 0.13-0.30 | 6-20 | 0.10-0.15 | 0.25-0.29 | 9.8-10. | 0.4-0.4 |
| Radówka | 12 | 8.91-9.45 | 8.01-9.31 | 225.2-231.0 | 0.0-0.1 | 0.001-0.002 | 0.34-0.42 | 2.8-4.3 | 0.21-0.94 | 0.26-0.35 | 4-8 | 0.19-0.36 | 0.39-0.40 | 2.2-2.3 | 0.23-0.2 |
| Pełknica | 13 | 7.04-9.95 | 7.95-9.36 | 253.0-258.0 | 0.0-0.2 | 0-0.012 | 0.32-0.38 | 3.6-4.0 | 0.23-0.53 | 0.30-0.41 | 2-9 | 0.18-0.29 | 0.28-0.31 | 3.9-4.3 | 0.1-0.2 |
| Tributary L. Pańskie | 14 | 6.07-7.30 | 7.87-9.15 | 285.0-373.0 | 0.1-0.2 | 0.001-0.004 | 0.45-1.40 | 4.6-4.9 | 0.37-0.88 | 0.31-0.43 | 5-16 | 0.00-0.07 | 0.05-0.22 | 3.1-4.8 | 0.2-0.4 |
| Drawica | 15 | 7.15-9.62 | 8.29-9.14 | 268.4-280.1 | 1.3-3.3 | 0.001-0.078 | 0.02-0.74 | 4.3-4.5 | 0.19-0.44 | 0.15-0.17 | 4-21 | 0.31-0.34 | 0.49-0.62 | 2.4-3.0 | 0.5-0.5 |
|  | 16 | 8.50-9.52 | 8.02-9.30 | 378.0-432.0 | 1.1-1.5 | 0.017-0.055 | 0.40-0.47 | 5.3-6.4 | 0.31-0.80 | 0.29-0.34 | 4-6 | 0.20-0.29 | 0.37-0.55 | 4.5-5.8 | 0.1-0.3 |
|  | 17 | 5.0-9.11 | 8.31-8.94 | 252.2-257.2 | 0.2-3.9 | 0.004-0.011 | 0.01-0.52 | 2.2-2.6 | 1.0-1.13 | 0.17-0.46 | 8-17 | 0.20-0.26 | 0.34-0.34 | 7.6-7.9 | 0.5-0.5 |
| Sitna | 18 | 6.9-8.28 | 8.51-8.90 | 377.2-406.3 | 0.1-0.6 | 0.001-0.011 | 0.08-0.30 | 1.9-2.9 | 0.37-0.74 | 0.17-0.19 | 7-25 | 0.22-0.29 | 0.36-0.48 | 2.8-3.0 | 0.9-0.3 |
|  | 19 | 8.45-9.28 | 8.65-8.93 | 374.1-403.2 | 0.2-0.5 | 0.003-0.008 | 0.09-0.32 | 2.9-4.7 | 0.27-0.54 | 0.11-0.18 | 8-27 | 0.19-0.30 | 0.32-0.50 | 2.6-3.1 | 0.3-0.3 |
| Prosta | 20 | 5.6-7.50 | 7.78-9.03 | 327-356.0 | 0.1-0.2 | 0.002-0.002 | 0.33-0.38 | 2.9-4.2 | 0.18-0.33 | 0.15-0.26 | 3-7 | 0.09-0.31 | 0.18-0.36 | 3.1-3.1 | 0.3-0.5 |
| Słopica | 21 | 7.26-9.24 | 8.4-9.03 | 213.1-225.3 | 0.2-3.4 | 0.009-0.014 | 0.05-0.43 | 2.5-3.3 | 4.49-0.50 | 0.26-0.33 | 10-27 | 0.17-0.25 | 0.25-0.32 | 6.4-7.4 | 0.3-0.4 |
|  | 22 | 8.15-10.23 | 8.54-8.99 | 221.7-229.6 | 0.3-3.7 | 0.008-0.014 | 0.03-0.35 | 3.7-7.7 | 0.52-0.88 | 0.15-0.22 | 2-10 | 0.31-0.38 | 0.47-0.66 | 4.9-5.2 | 0.2-0.3 |
| Korytnica | 23 | 10.5-11.11 | 8.11-9.59 | 296.0-346 | 0.1-0.4 | 0.005-0.007 | 0.30-0.36 | 4.1-5.0 | 0.41-0.68 | 0.19-0.32 | 4-6 | 0.26-0.30 | 0.48-0.55 | 5.2-6.5 | 0.5-0.5 |
|  | 24 | 7.7-10.66 | 8.17-9.54 | 291.0347- | 0.2-0.2 | 0.06-0.010 | 0.33-0.39 | 3.8-4.9 | 0.38-0.65 | 0.21-0.36 | 5-9 | 0.27-0.27 | 0.44-0.63 | 4.70-6.2 | 0.5-0.7 |
|  | 25 | 9.23-9.25 | 8.59-9.18 | 246.1-294.8 | 0.2-1.1 | 0.009-0.013 | 0.07-0.34 | 1.0-2.4 | 0.62-0.62 | 0.26-0.43 | 1-27 | 0.08-0.25 | 0.48-0.68 | 9.6-11.3 | 0.6-0.7 |
|  | 26 | 8.67-9.58 | 8.71-9.42 | 245.3-253.5 | 0.2-1.4 | 0.005-0.017 | 0.06-0.33 | 0.7-2 | 0.36-0.62 | 0.08-0.34 | 3-16 | 0.34-0.45 | 0.70-0.90 | 12.3-14.6 | 0.3-0.5 |
| Płociczna | 27 | 9.8-9.99 | 8.08-9.37 | 300.3-361 | 0.7-1.1 | 0.016-0.017 | 0.34-0.54 | 4.1-5.9 | 0.41-0.54 | 0.12-0.24 | 7-12 | 0.04-0.19 | 0.37-0.51 | 3.6-5.2 | 0.5-0.5 |
|  | 28 | 8.62-9.89 | 8.20-9.46 | 262.2-263-7 | 0.5-1.9 | 0.009-0.020 | 0.07-0.41 | 2-2.3 | 0.23-0.77 | 0.11-0.3 | 4-9 | 0.27-0.34 | 0.43-0.69 | 10.2-10.4 | 0.5-0.6 |
|  | 29 | 7.75-10.50 | 8.61-9.53 | 260.7-269 | 0.2-1.4 | 0.007-0.008 | 0.05-0.41 | 0.8-2.4 | 0.42-2.75 | 0.21-0.24 | 1-12 | 0.22-0.39 | 0.60-0.67 | 14-15.4 | 0.3-0.4 |
| Młynówka | 30 | 5.9-9.10 | 8.28-9.34 | 244.4-257 | 0-1.7 | 0.003-0.008 | 0.02-0.55 | 0.2-2 | 0.30-1.06 | 0.10-0.39 | 3-13 | 0.13-0.15 | 0.22-0.34 | 6.8-7.2 | 0.5-0.6 |
| Cieszynka | 31 | 7.32-9.51 | 8.51-9.52 | 254.1-256.9 | 0.1-0.7 | 0.006-0.013 | 0.02-0.46 | 0.7-3.1 | 0.20-1.76 | 0.30-0.41 | 2-7 | 0.14-0.17 | 0.41-0.44 | 7.9-7.9 | 0.7-0.7 |
| Szczuczna | 32 | 8.72-9.62 | 8.41-9.07 | 246.5-249.2 | 0-0.9 | 0.001-0.004 | 0.08-0.32 | 1-3.2 | 0.36-0.4 | 0.17-0.32 | 12-12 | 0.14-0.28 | 0.35-0.36 | 4.5-5.0 | 0.3-0.4 |

**Table S3**

Rare, threatened, and protected species of Mollusk of rivers with different types of catchments. *- according to The IUCN Red List of Threatened Species <https://www.iucnredlist.org/> and Cuttelod A., Seddon M., Neubert E. 2011. European Red List of Non-marine Molluscs. Publications Office of the European Union, Luxembourg; ** Polish Red List of Species: Polish Red List of Species: Głowaciński, Z., Nowacki, J. (Eds). 2004. Polish Red Data Book of Animals. Invertebrates. Instytut Ochrony Przyrody PAN, Akademia Rolnicza im. A. Cieszkowskiego, Kraków-Poznań, 447 pp; ***according to the Polish Journal of Laws 2016, item 2183. Regulation of the Minister of the Environment of December 16, 2016, on the protection of animal species: EN - endangered species, LC- least concern species, VU- vulnerable species, NA- not applicable; ****- species listed at EU Habitats Directive, Council Directive 92/43/EEC (1992), Annexes II and IV.

| Taxon | IUCN category  globally* | | IUCN category in Europe* | Population trend* | | Polish Red List** | Species protection***  1-total  2-partial | | Type of species |
| --- | --- | --- | --- | --- | --- | --- | --- | --- | --- |
|  |  |  |  | Global | Europe |  |  |  |  |
| **Freshwater bivalves** | |  | | |  | | | | |
| *Unio pictorum* | LC | | LC | unknown | unknown | - | - | Native | |
| *Unio tumidus* | LC | | LC | unknown | unknown | - | - | Native | |
| *Unio crassus ***** | EN | | VU | decreasing | decreasing | EN | 1 | Native | |
| *Anodonta anatina* | LC | | LC | decreasing | unknown | - | - | Native | |
| *Anodonta cygnea* | LC | | NT | decreasing | decreasing | EN | 2 | Native | |
| *Musculium lacustre* | LC | | LC | unknown | unknown | - | - | Native | |
| *Sphaerium corneum* | LC | | LC | unknown | unknown | - | - | Native | |
| *Dreissena polymoprha* | LC | | NA | increasing | increasing | - | - | Alien, invasive | |
| **Freshwater snails** | |  | | |  | | | | |
| *Ancylus fluviatilis* | LC | | LC | unknown | unknown | - | - | Native | |
| *Acroloxus lacustris* | LC | | LC | stable | stable | - | - | Native | |
| *Theodoxus fluviatilis* | LC | | LC | stable | unknown | - | - | Native | |
| *Bithynia tentaculata* | LC | | LC | stable | stable | - | - | Native | |
| *Stagnicola corvus* | LC | | LC | unknown | unknown | - | - | Native | |
| *Radix auricularia* | LC | | LC | stable | stable | - | - | Native | |
| *Potamopyrgus antipodarum* | LC | | NA | stable | increasing | - | - | Alien, invasive | |
| *Viviparus contectus* | LC | | LC | decreasing | decreasing | - | - | Native | |
| *Valvata cristata* | LC | | LC | unknown | unknown | - | - | Native | |
| *Anisus vortex* | - | | LC | unknown | unknown | - | - | Native | |
| *Planorbis planorbis* | LC | | LC | unknown | stable | - | - | Native | |

**Table S4**

Summary of the canonical correspondence analysis (CCA) carried out on freshwater

mollusc species and environmental data in studied rivers.

| Axes | 1 | 2 | 3 | 4 |
| --- | --- | --- | --- | --- |
| Eigenvalues: | 0.325 | 0.193 | 0.151 | 0.118 |
| Species-environment correlations: | 0.942 | 0.925 | 0.894 | 0.918 |
| Cumulative percentage variance |  |  |  |  |
| of species data: | 17.9 | 28.5 | 36.8 | 43.3 |
| of species-environment relation: | 26.8 | 42.7 | 55.1 | 64.8 |
|  | | | | |
| Monte Carlo Permutation test of significance of CCA axes: | | | | |
| axis 1 | F = 3.933; p = 0.0020 | | | |
| all axis | F = 2.793; p = 0.0020 | | | |
